# The Responses of Germ-Free Zebrafish (*Danio rerio*) to Varying Bacterial Concentrations, Colonization Time Points, and Exposure Duration

**DOI:** 10.3389/fmicb.2019.02156

**Published:** 2019-09-18

**Authors:** Fang Tan, Samwel Mchele Limbu, Ye Qian, Fang Qiao, Zhen-Yu Du, Meiling Zhang

**Affiliations:** ^1^Laboratory of Aquaculture Nutrition and Environmental Health, School of Life Sciences, East China Normal University, Shanghai, China; ^2^Department of Aquatic Sciences and Fisheries Technology, University of Dar es Salaam, Dar es Salaam, Tanzania

**Keywords:** germ-free zebrafish, colonization conditions, host responses, gnotobiotic zebrafish, host microbiota, mono-association

## Abstract

Colonizing germ-free (GF) zebrafish with specific bacterial species provides the possibility of understanding the influence on host biological processes including gene expression, development, immunity, and behavioral responses. It also enlightens our understanding on the host-microbe interactions within the physiological context of a living host. However, the responses of GF zebrafish to various colonization conditions such as bacterial concentrations, colonization time points, and exposure duration remain unclear. To address this issue, we explored the responses of GF zebrafish by using two bacterial species at varying concentrations, colonization time points and exposure duration. Therefore, we mono-associated GF zebrafish with *Escherichia coli* DH5α or *Bacillus subtilis* WB800N at concentrations ranging from 10^2^ to 10^7^ CFU/ml either at 3 day post fertilization (dpf) or 5 dpf for 24 or 48 h. We evaluated the responses of GF zebrafish by analyzing the survival rate, colonization efficiency, nutrients metabolism, intestinal cell proliferation, innate immunity, stress, and behavior responses by comparing it to conventionally raised zebrafish (CONR) and GF zebrafish. The results indicated that the final bacteria concentrations ranging from 10^2^ to 10^4^ CFU/ml did not cause any mortality when GF mono-associated larvae were exposed to either *E. coli* DH5α or *B. subtilis* WB800N at 3 or 5 dpf, while concentrations ranging from 10^6^ to 10^7^ CFU/ml increased the mortality, particularly for 5 dpf owing to the decrease in dissolved oxygen level. The *E. coli* DH5α mainly induced the expression of genes related to nutrients metabolism, cell proliferation and immunity, while *B. subtilis* WB800N mainly upregulated the expression of genes related to immunity and stress responses. Moreover, our data revealed that GF zebrafish showed higher levels of physical activity than CONR and the microbial colonization reduced the hyperactivity of GF zebrafish, suggesting colonization of bacteria affected behavior characteristics. This study provides useful information on bacterial colonization of GF zebrafish and the interaction between the host and microbiota.

## Introduction

The microbiota residing within the animal gastrointestinal tract play important roles in various physiological processes, including modulation of immune system (Koch et al., 2018), enhancement of intestinal barrier (Takiishi et al., 2017), absorption or digestion of nutrients (Delzenne and Singer, 2016), inhibition of pathogens (Qin et al., 2018) and host behavior and neurodevelopment (Diaz Heijtz et al., 2011; Davis et al., 2016; Phelps et al., 2017). Therefore, studies on the relationships between the host and intestinal microbiota have received more attentions in recent years (Zhao, 2013). However, the causation relationships between intestinal microbiota functions and host responses in these animals are difficult to establish because the gut microbes and the animals are composed of incredibly complex networks and are subject to multiple influences from various factors (Williams, 2014; Rolig et al., 2015). In order to understand the exact functions of commensals, causation studies are required as opposed to the current correlations approaches (Zhao, 2013). Germ-free (GF) or gnotobiotic animals act as simple and powerful tools for elucidating the causality relationships between hosts and their microbial residents (Gordon, 1960; Pham et al., 2008; Williams, 2014). Various GF animal models including mice, rats, guinea pigs, goats, platyfish (*Xiphophorus maculatus*) and chickens have been established (Shaw, 1957; Gordon, 1960; Pham et al., 2008). These models have broadened our understanding on microbiota and host interactions in a variety of animals (Gordon, 1960; Shimizu et al., 1998; Pham et al., 2008; Melancon et al., 2017). However, the production process of these gnotobiotic animals is technically challenging, especially for big animals such as pigs and goats. Furthermore, the long reproductive cycles of big animals require longer experimental periods and maintaining them in sterile conditions is difficult, which limits the wide application of these GF animal models (Li et al., 2014a).

The GF zebrafish (*Danio rerio*) model was successfully established in 2004 (Rawls et al., 2004). The GF zebrafish provides a number of advantages that simplify the generation of gnotobiotic organisms and research the interaction between the host and microbiota. First, zebrafish have transparent larvae (from time of fertilization to early adulthood), which are convenient to perform *in vivo* observations of intestinal bacteria (Rawls et al., 2007). Second, zebrafish have high fertility (200–300 eggs each female zebrafish) such that eggs are spawned throughout the year typically every 4 to 7 days after attaining sexual maturity (around 3–4 month) under normal experimental conditions (Clelland and Peng, 2009). Third, zebrafish larvae can be easily raised under laboratory conditions (Ribas and Piferrer, 2014). These attributes, combined with extensive homologies between zebrafish and mammals at genomic, anatomical and physiological levels, and cell types including absorptive enterocytes and secretory goblet cells and enteroendocrine cells allow GF zebrafish to serve as powerful models for revealing the functions of intestinal microbiota (Ng et al., 2005; Pham et al., 2008; Brugman, 2016). Accordingly, methods for production and colonization of GF zebrafish have been established (Al-Asmakh et al., 2012). Previous studies indicated that, it is more practical to colonize fish with a single bacterial species (mono-association) in order to study the effects of a specific bacteria on the host rather than colonizing GF fish with a complex microbiota (conventionalization) communities (Galindo-Villegas et al., 2012; Semova et al., 2012). Consequently, considering its practicality and simplicity, mono-association of zebrafish is widely applied to screen and evaluate the functions of probiotics and pathogens *in vivo* (Ran et al., 2018). Moreover, mono-association of zebrafish is also used to study the influence of bacterial colonization on host biological processes (Rendueles et al., 2012; Ran et al., 2018).

However, the mono-association conditions including the inoculation concentrations, inoculation time points and exposure time for GF zebrafish vary considerably among studies. For instance, mono-associated zebrafish were generated by adding *Aeromonas veronii* into the culture medium of 5 dpf GF larvae to a final concentration of 10^6^ CFU/ml (Bates et al., 2006). However, *Escherichia coli* K46 were added into culture media of 5 dpf GF larvae to a final concentration of 10^3^ CFU/ml for 3 days (Kjelstrup et al., 2017). On the contrary, 4 dpf larval fish were exposed to a density of approximately 10^6^ CFU/ml for 24 h (Robinson et al., 2018). Moreover, Siriyappagouder et al. (2018) exposed GF zebrafish larvae (2 dpf) to 2 × 10^5^ CFU/ml *Debaryomyces* sp. or *Pseudozyma* sp. for 24 h. Nevertheless, whether these mono-association conditions are sufficient to induce host reactions or cause damages have not been thoroughly investigated. More specifically, the effects of different bacterial concentrations, colonization time points and duration of exposure on the host responses are still unknown.

In the present study, we colonized GF zebrafish with *E. coli* DH5α or *Bacillus subtilis* WB800N expressing green fluorescent protein (GFP) as a biomarker and compared them with conventionally raised zebrafish (CONR) zebrafish and GF zebrafish. The *E. coli* and *B. subtilis* are common commensals in the fish gut (Munro et al., 1995; Rawls et al., 2006; Rekecki et al., 2012; Li et al., 2014b) and are easy for fluorescence labeling. We then evaluated colonization efficiency and the host responses in nutrients metabolism, innate immunity, cell proliferation, stress, and behavior characteristics under varying bacterial mono-association conditions.

## Materials and Methods

### Ethics Statement

All experiments were performed under the guidance for the care and use of laboratory animals in China. This research was approved by the Committee on the Ethics of Animal Experiments of East China Normal University (ECNU) (No. F20140101), Shanghai, China.

### Zebrafish Husbandry

Wild type male and female adults (4 to 6 months) zebrafish line AB were obtained from the Chinese National Zebrafish Resource Center (Wuhan, China) and maintained at the Laboratory of Aquaculture Nutrition and Environmental Health (LANEH) of ECNU, Shanghai, China. All the parents fish were fed on a commercial diet (Shengsuo, Yantai, China) containing 50% protein and 8% lipid twice a day. The water temperature was maintained at 25 to 27°C. The fish were maintained under a 14 h day and 10 h dark photoperiod consistent with the standard culture conditions for zebrafish care (Lu et al., 2019).

### Production of Conventionally Raised (CONR) and Germ-Free (GF) Zebrafish

Natural bred eggs were collected immediately after hatching and transferred into a normal zebrafish culture medium. The CONR embryos were hatched in unsterilized gnotobiotic zebrafish medium (GZM), which contained 0.06 mg/ml marine salt (Hai Ye, Shanghai, China). The hatched larvae were continually reared in the GZM (unsterilized) at 28°C under a constant photoperiod cycle of 14 h light and 10 h dark with an external PRX-80 Intelligent Incubator System (Sai Fu, Ningbo, China). Fresh media were replaced every day.

The production of GF zebrafish was performed as previously reported (Pham et al., 2008) with some modifications. Briefly, the embryos obtained from adult parents zebrafish were washed three times by using sterile water (3 min per time at room temperature) and incubated at 28°C for 6 h in 50 ml of antibiotic-gnotobiotic zebrafish medium (AB-GZM), which contained 0.06 mg/ml marine salt (Hai Ye, Shanghai, China), 100 μg/ml ampicillin (Yeasen, Shanghai, China), 5 μg/ml kanamycin (Yeasen, Shanghai, China), and 250 ng/ml amphotericin B (Yeasen, Shanghai, China). Afterward, the embryos were soaked into 0.04% polyvinylpyrrolidone (Sangon Biotech, Shanghai, China) solution for 40 s and washed three times by using the sterile GZM. Thereafter, the embryos were further soaked into 0.003% sodium hypochlorite (Sangon Biotech, Shanghai, China) for 15 min and washed three times by using the sterile GZM (autoclaved) as reported previously (Ran et al., 2016). The embryos were reared in 6-well sterile cell culture plates and immersed into the sterile GZM at a density of approximately two individual per 5 ml water. All culture processes were conducted at 28°C under a constant photoperiod cycle of 14 h light and 10 h dark with an external PRX-80 Intelligent Incubator System (Sai Fu, Ningbo, China). Fifty percent by volume (50%) of GZM in each well was replaced with fresh sterile medium every day to avoid waste accumulation and dissolved oxygen limitation as reported previously (Sheng et al., 2018). The collecting GF fish media were cultured aerobically and anaerobically on a daily basis using a tryptic soy agar (TSA) plate and Luria-Bertani (LB) plate at 38°C for at least 48 h to detect any bacteria that may be able to grow on the plates (Rawls et al., 2004).

### Labeling of Bacteria by Using Green Fluorescent Protein (GFP)

#### Bacterial Strains and Culture Conditions

Two bacterial strains, *E. coli* DH5α and *B. subtilis* WB800N were used in the present study. We used *E. coli* and *B. subtilis* because they have been identified previously from the gut of healthy fish (Munro et al., 1995; Rekecki et al., 2012). The *E. coli* DH5α labeled by GFP was obtained as follows. The recombinant plasmid pET-28a-c(+) (Novagen, Malaysia) containing a EGDe-gfp gene and a kanamycin resistance gene was integrated into a specific target location of *E. coli* DH5α chromosome. Subsequently, bacteria were cultured in LB liquid media supplemented with 50 ug/ml kanamycin (Sigma Chemical, Co., St. Louis, MO, United States) at 37°C for 14 h with shaking at 190 rpm/min. A 1 μM Isopropyl β-D-1-thiogalactopyranoside (IPTG) (Sigma Chemical, Co., St. Louis, MO, United States) was added to induce the expression of GFP when the bacteria OD_600_ culture reached between 0.6 and 0.8 (Wiles et al., 2018). The GFP labeled *B. subtilis* WB800N was obtained as follows. The recombinant plasmid pHT01 (BioVector NTCC, Inc., Beijing, China) containing a EGDe-gfp gene and a chloramphenicol resistance gene was integrated into a specific target location of *B. subtilis* WB800N chromosome. The *B. subtilis* WB800N were cultured in LB liquid media with the presence of 5 ug/ml chloramphenicol (Sigma Chemical, Co., St. Louis, MO, United States) at 37°C for 24 h with shaking at 170 rpm/min. The expression of GFP was induced as described above for *E. coli*.

### Production of GF Mono-Associated Zebrafish

The bacterial culture was centrifuged at 7500 rpm for 10 min to remove the supernatant from the medium. The cell pellet was washed three times in sterile water. Then it was resuspended with sterile GZM and transferred to the GF zebrafish medium at a final concentrations ranging from 10^2^ to 10^7^ CFU/ml. The GF zebrafish larvae at 3 and 5 dpf were transferred into another six-well sterile plates (10 fish each well) by using sterile pipettes. We selected the 3 dpf larvae because 3 dpf is the developmental stage at which CONVR fish hatch from their chorions and it is the first time to be colonized by microbiota. The 5 dpf is the time when zebrafish begin to feed after their yolk has been exhausted (Pham et al., 2008). The GF zebrafish larvae were aseptically divided into two treatment groups; GF and GF mono-associated. The GF mono-associated were obtained by exposing GF zebrafish larvae to *E. coli* DH5α at concentrations ranging from 10^2^ to 10^7^ CFU/ml or *B. subtilis* WB800N at concentrations ranging from 10^2^ to 10^6^ CFU/ml for 24 or 48 h at 28°C.

The GF zebrafish larvae exposed to *E. coli* DH5α at final concentrations of 10^2^, 10^4^, 10^5^, 10^6^, and 10^7^ CFU/ml are hereby referred to as 2E, 4E, 5E, 6E, and 7E groups respectively, while the GF zebrafish exposed to *B. subtilis* WB800N at final concentrations of 10^2^, 10^4^, 10^5^, and 10^6^CFU/ml are termed as 2B, 4B, 5B, and 6B groups, respectively. We used two incubation periods of 24 and 48 h because they are commonly used in GF studies (Ding et al., 2018; Schlomann et al., 2018). After the incubation, the larvae from each group were washed five times with the sterile GZM to remove the bacteria adhered to the surface and then photographed by fluorescent microscope (SZX16, Olympus, Tokyo, Japan) to detect the existence of the two bacterial strains in the gastrointestinal tract.

In order to quantify the number of colonized bacteria in each fish, 10 zebrafish larvae were transferred into 1.5 ml sterile tubes and washed 10 times with sterile water to remove the bacteria adhered to the surface. Afterward, zebrafish were homogenized by using sterile glass beads (600 mm) and 500 ml of autoclaved PBS (1x) by using FastPrep Cell Disrupter (BIO101/FP120 QBioGene) at 60 Hz for 30 s to release bacteria inside the body. Serial dilutions of recovered suspensions were spotted on LB plates containing kanamycin to detect *E. coli* DH5α expressing GFP specifically and containing chloramphenicol where only *B. subtilis* WB800N expressing GFP can grow.

At 4 or 5 dpf, the dissolved oxygen level in the culture media of GF zebrafish and the mono-associated zebrafish treated with the two bacteria at concentrations ranging from 10^2^ to 10^8^ CFU/ml for 24 or 48 h was measured by using a portable dissolved oxygen meter (JPB-607A, Rex, Shanghai, China).

### Isolation of RNA, Synthesis of cDNA, and Quantitative PCR (qPCR)

We determined the mRNA expression levels of genes related to nutrients metabolism, innate immune, cell proliferation, stress responses, and behavior responses. Six larvae samples from each treatment (each sample containing 20 larvae) were collected and homogenized in 1000 μL lysis buffer. The total RNA was extracted by using a Tri Pure Reagent (Aidlab, Beijing, China). The quality and quantity of total RNA were tested by Nanodrop 2000 Spectrophotometer and electrophoresis (Thermo Fisher Scientific, Waltham, MA, United States). The RNAs having an A260/A280 absorbance ratio of 1.8 to 2.0 and an A260/A230 ratio of > 2.0 were used for subsequent analyses. The cDNAs were synthesized by using 1000 ng total RNA as the template by utilizing a PrimerScript^TM^ RT reagent Kit (RR047A, Takara, Shiga, Japan) according to the manufacturer’s instructions. Quantitative reverse transcription PCR (qPCR) (20 μL) containing 10 μL of 2 × SYBR qPCR Mixture (Aidlab, Beijing, China), 100 ng cDNA, 300 nM of qPCR primers, and 6.4 μL nuclease-free water was performed in a CFX Connect Real-Time System (Bio-Rad, Hercules, CA, United States). The qPCR reaction was conducted at 95°C for 10 min, 40 cycles of 95°C for 5 s and 60°C for 18 s. Melting curves of amplified products were generated to ensure the specificity of assays at the end of each qPCR. The primer sequences used for qPCR in the present study are shown in Supplementary Table S1. Elongation factor 1 α (*ef1*α), β-actin, and Glyceraldehyde-3-phosphate dehydrogenase (*gapdh*) were used as the reference genes. The relative mRNA expression levels of genes were estimated by using the method of 2^–ΔΔ^ *^Ct^* thereof, ΔCt = Ct_target_ − (Ct_EF__1__α_ + Ct_β–actin_ + Ct_gapdh_)/3.

### Behavioral Studies

The zebrafish larvae from six treatments (GF, CONR, 4E, 6E, 4B, and 6B) were used for behavioral responses experiment. The zebrafish from each group were transferred carefully from 6-well plate into the 48-well plate by using sterile pipettes. During this study, each 48-well plate contained 1 ml GZM and 2 ml paramecium solution to serve as food. The plate was then placed into the behavior detector (ViewPoint, Life Sciences, Germany) to record activities of zebrafish larvae. Locomotor activities were recorded every minute for a period of 48 h. The speed was set as lower than 8 mm/s for slow motion, from 8 to 20 mm/s for the medium speed and higher than 20 mm/s for fast speed. All behavioral tests were performed in a controlled room at 28.5°C. The room was humidified to minimize the evaporation of the water in the testing wells. This experiment was repeated three times.

### Statistical Analyses

Results are presented as mean ± SEM whenever applicable and data were tested for normality by using Shapiro–Wilk test and homoscedasticity by using Levene’s test. Most of the measured parameters were normally distributed, while *state3* and *tph1a* for *E. coli* and *bf* and *myd88* for *B. subtilis* were not normally distributed even after log transformation (Supplementary Table S2). One-way analysis of variance (ANOVA) was used to compare the responses of normally distributed data for CONR, GF and mono-associated zebrafish at different concentrations. Dunnett’s multiple comparisons test was used to specify specific differences when ANOVA indicated statistical significance. The Kruskal–Wallis (H) test was used to compare non-distributed data followed by the Mann–Whitney (*U*) test for pairwise comparisons. All statistical analyzes were performed by using the Statistical Package for the Social Sciences (SPSS) version 20.0 software for windows (IBM, Armonk, NY, United States). Results with *p*-value < 0.05 were considered statistically significant.

## Results

### The Survival Rate and the Bacterial Colonization Efficiency of the GF Mono-Associated Zebrafish With *E. coli* DH5α at 3 and 5 dpf

**FIGURE 1:**
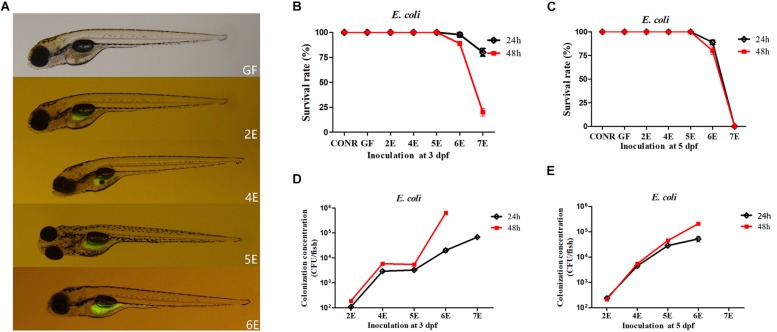
Germ-free zebrafish colonized with GFP labeled *Escherichia coli* DH5α. **(A)** Fluorescence images of GF zebrafish and GF zebrafish mono-associated with 10^2^, 10^4^, 10^5^, 10^6^ CFU/ml *E. coli* DH5α at 3 dpf for 24 h. The magnification of the fluorescence microscope is 3.5×. **(B)** The survival rate of germ-free (GF) zebrafish, conventional raised (CONR) zebrafish and germ-free zebrafish exposed to different concentrations of *E. coli* DH5α at 3 dpf for 24 or 48 h. **(C)** The survival rate of germ-free (GF) zebrafish, conventionally raised (CONR) zebrafish and germ-free zebrafish exposed to different concentrations of *E. coli* DH5α at 5 dpf for 24 or 48 h. **(D)** Colonization concentrations (CFU/fish) of mono-associated zebrafish colonized at 3 dpf for 24 or 48 h. **(E)** Colonization concentrations (CFU/fish) of mono-associated zebrafish at 5 dpf for 24 or 48 h. All data **(B–E)** are presented as means ± SEM, *n* = 45 per group.

The GF zebrafish at 3 dpf were colonized with different concentrations of GFP labeled *E. coli* DH5α and immersed for 24 or 48 h. The photographs showed that the fluorescent bacteria successfully colonized the gastrointestinal tract of zebrafish (4 dpf) instead of other tissues (Figure 1A). The survival rate of mono-associated larvae were also determined. The results indicated that, the survival rate of 2E, 4E, and 5E zebrafish remained 100% at 3 dpf (Figure 1B). However, when 6E zebrafish were exposed for 24 h, the survival rate decreased to 93.3% (Figure 1B). The survival rate further decreased to 86.3% when the exposure time was increased to 48 h (Figure 1B). The survival rate of 7E decreased to 73.3% after 24 h and 26.7% when the exposure time was increased to 48 h (Figure 1B).

The survival rate of GF zebrafish exposed to *E. coli* DH5α at 5 dpf were also detected. The results indicated that, the survival rate of zebrafish remained 100% from 2E to 5E (Figure 1C). When 6E zebrafish were exposed for 24and 48 h, the survival rate decreased to 93.3 and 73.4%, respectively. The exposure time did not exert significant differences in the survival rate except for 6E group (Figure 1C).

In order to identify the possible reason for the mortality caused by higher bacteria colonization concentrations, the dissolved oxygen content in the zebrafish media was detected. The results indicated that, the higher bacterial inoculation densities (more than 10^6^CFU/ml *E. coli* DH5α) reduced the dissolved oxygen level in the media (Supplementary Figure S1A). The expression level of hypoxia-inducible factors 3α (*hif3*α) was also detected in these treatments. The expression level of *hif3*α increased significantly in 7E and 8E groups (Supplementary Figure S1B) consistent with the results on lower dissolved oxygen level. These results suggested that, higher concentrations of bacteria colonization may cause death of fish due to the lack of dissolved oxygen in the media.

To understand the bacterial colonization efficiency under different mono-association densities and inoculation time points, the colonized bacteria numbers of each fish were counted. For GF inoculated at 3 dpf, we found that when 10^2^ and 10^4^CFU/ml of bacteria (2E and 4E) were inoculated, the detected colonized bacterial quantities were 10^2^ and 10^4^CFU/fish (Figure 1D). The concentration of colonized bacteria did not increase when zebrafish were exposed to 10^5^ CFU/ml *E. coli* DH5α (5E). When the inoculation concentrations were increased to 10^6^ and 10^7^ CFU/ml (6E and 7E) for 24 h, the colonized bacterial levels were around 10^4^ and 10^5^ CFU/fish, but when the exposure time was increased to 48 h, the quantities of colonized bacteria increased (Figure 1D). For GF inoculated at 5 dpf, the exposure time did not exert significant differences in colonization concentrations, while the colonization concentration with bacteria at 6E for 48 h increased significantly than 24 h (Figure 1E).

### The Survival Rate and the Bacterial Colonization Efficiency of the GF Mono-Associated Zebrafish With *B. subtilis* WB800N at 3 and 5 dpf

**FIGURE 2:**
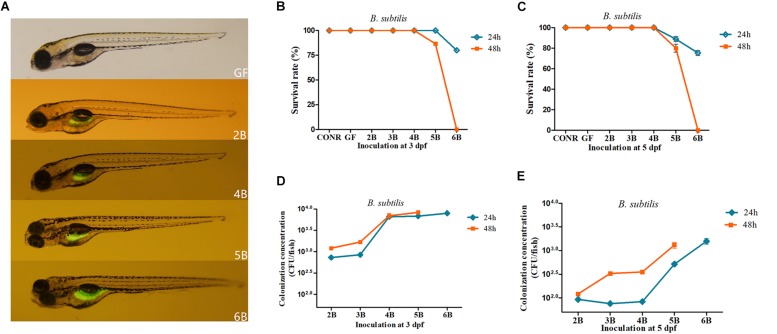
Germ-free zebrafish colonized with GFP labeled *Bacillus subtilis* WB800N. **(A)** Fluorescence images of germ-free (GF) zebrafish and germ-free (GF) zebrafish mono-associated with different concentrations of *B. subtilis* WB800N (10^2^, 10^4^, 10^5^, 10^6^CFU/ml). The magnification of the fluorescence microscope is 3.5×. **(B)** The survival rate of germ-free (GF) zebrafish, conventional raised (CONR) zebrafish and germ-free zebrafish exposed to different concentrations of *B. subtilis* WB800N at 3 dpf for 24 or 48 h. **(C)** The survival rate of GF zebrafish, conventionally raised (CONR) zebrafish and germ-free zebrafish exposed to different concentrations of *B. subtilis* WB800N at 5 dpf for 24 or 48 h. **(D)** Colonization concentrations (CFU/fish) of mono-association zebrafish colonized at 3 dpf for 24 or 48 h. **(E)** Colonization concentrations (CFU/fish) of GF zebrafish at 5 dpf for 24 or 48 h. All data **(B–E)** are presented as means ± SEM, *n* = 45 per group.

Similar to the previous results, we first detected whether GFP-labeled *B. subtilis* WB800N reached the intestinal tract of GF zebrafish. The results indicated that, the fluorescent bacteria colonized successfully the gastrointestinal tract of the GF zebrafish (4 dpf) after inoculation with GFP-labeled *B. subtilis* WB800N at different concentrations ranging from 10^2^ to 10^6^ CFU/ml (2B, 4B, 5B, 6B) at 3 dpf for 24 h (Figure 2A). We further determined the survival rate of the GF larvae after incubation with *B. subtilis* WB800N at 3 dpf for 24 or 48 h. The survival rate for 2B, 3B, and 4B at 3 dpf remained 100% (Figure 2B). When GF zebrafish were exposed to 10^5^ CFU/ml of *B. subtilis* WB800N for 48 h, the survival rate of 5B decreased to 90%. When the concentration was increased to 10^6^ CFU/ml for 24 h, the survival rate of 6B further declined to 80%, and when the exposure time was increased to 48 h, all the zebrafish died (Figure 2B).

When GF were mono-associated with *B. subtilis* WB800N at 5 dpf, they had lower survival rate than 3 dpf. Similar to previous results, the survival rate of 2B, 3B, 4B mono-associated zebrafish for 24 h was 100% (Figure 2C). However, the survival rate of 5B, 6B mono-associated zebrafish was reduced to 89% and 77.5%, respectively. The survival rate of 5B mono-associated zebrafish decreased to 81.6% when the exposure time was increased to 48 h, while it was zero (all the fish died) for 6B mono-associated zebrafish (Figure 2C), suggesting that inoculation with 10^6^ CFU/ml of *B. subtilis* WB800N or higher densities caused severe mortality to the mono-associate zebrafish.

Accordingly, the dissolved oxygen level of zebrafish media and the expression level of *hif3*α were also assessed. Similar to the above results, the higher inoculation densities of *B. subtilis* WB800N especially 10^7^ and 10^8^ CFU/ml decreased drastically the dissolved oxygen level in the media (Supplementary Figure S2A) and increased the transcriptional level of *hif3*α (Supplementary Figure S2B). The inoculation concentrations ranging from 10^2^ to 10^6^ CFU/mL did not influence dissolved oxygen concentration in zebrafish media (Supplementary Figure S2A).

The colonized bacteria were around 10^3^ to 10^4^ CFU/fish when the inoculation levels of *B. subtilis* WB800N increased from 10^2^ to 10^4^ CFU/ml (2B, 3B, and 4B) at 3 dpf (Figure 2D). Interestingly, the colonized bacterial level was around 10^4^ CFU/fish even when the inoculation concentrations reached 10^5^ and 10^6^ CFU/ml (5B, 6B) for 24 h (Figure 2D). Extending the exposure time from 24 to 48 h increased the colonization concentrations of each fish. The final colonized bacteria of 2B, 3B, 4B, 5B, and 6B groups for 24 h ranged from 10^2^ to 10^3^ CFU/fish and the bacterial colonization quantities increased when the exposure time was prolonged (Figure 2E).

### The Host Responses to Different Concentrations of *E. coli* DH5α

In order to understand the host responses to different colonization conditions, the mRNA expression of genes related to nutrients metabolism, innate immunity, stress responses, and cell proliferation were measured according to the previous studies (Rawls et al., 2006; Ran et al., 2016). The responses were analyzed among mono-associated zebrafish exposed to different concentrations of *E. coli* DH5α, GF zebrafish and CONR zebrafish at 4 dpf.

**FIGURE 3:**
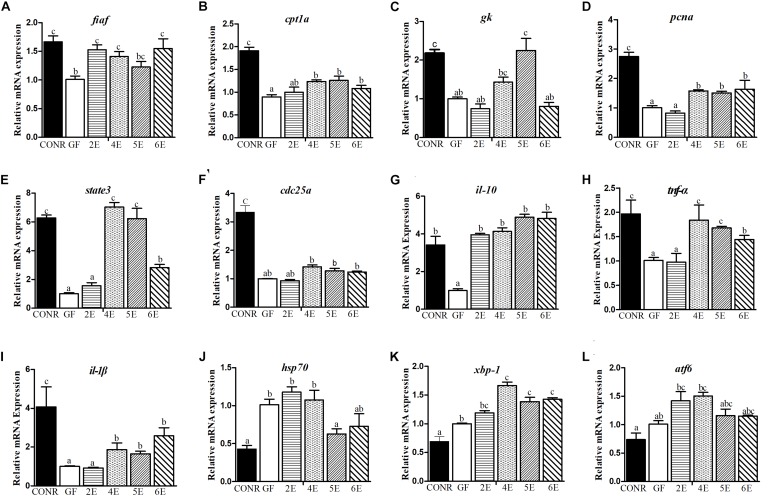
Comparisons of gene expression levels among germ-free (GF) zebrafish, CONR zebrafish and mono-associated (GF) zebrafish with different concentrations of *E. coli* DH5α. **(A)** The expression levels of nutrients metabolism related genes *fiaf*, **(B)** *cpt1a*, **(C)** *gk.* **(D)** The expression level of cell proliferation related genes, *pcna*, **(E)** *state3*, **(F)** *cdc25a.* **(G)** The expression levels of innate immune related genes *il-10*, **(H)** *tnf-*α and **(I)** *il-1*β. **(J)** The expression levels of damage stress related genes *hsp70*, **(K)** *xbp1* and **(L)** *atf6.* The results were shown as mean ± SEM of three independent experiments with six replicates for each experiment, *n* = 90, per group. Different letters indicate statistically significant differences among groups (*p* < 0.05).

In general, CONR zebrafish and mono-associated zebrafish upregulated the three measured nutrients metabolism related genes than GF zebrafish. Bacterial colonization enhanced the expression level of fasting-inducing adipose factor (*fiaf*) gene than GF zebrafish except 5E (Figure 3A). Most mono-associated zebrafish elevated the carnitine palmitoyltransferase 1a (*cpt1a*) gene expression compared with GF (Figure 3B). Additionally, mono-associated zebrafish had higher glucokinase (*gk*) gene expression than GF zebrafish except for 2E and 6E group (Figure 3C).

Furthermore, the three cell proliferation related genes measured in the mono-associated zebrafish were all activated relative to GF zebrafish. The mono-associated zebrafish at 4E, 5E, and 6E had higher proliferating cell nuclear antigen (*pcna*; Figure 3D) and signal transducer and activator of transcription 3 (*state3*; Figure 3E) expression levels than GF zebrafish. Moreover, with exceptional of the lowest colonization concentration (2E), the other mono-associated zebrafish upregulated slightly the expression of cell division cycle 25A (*cdc25a*) gene than GF zebrafish (Figure 3F).

The transcription levels of the six immunity cytokines were measured to determine the effects of bacteria exposure on host immune response. The transcription levels of interleukin-10 (*il-10*) were markedly promoted among larvae treated with *E. coli* DH5α relative to GF zebrafish (Figure 3G). Furthermore, with the exceptional of the 2E mono-association concentration, the transcription levels of tumor necrosis factor alpha (*tnf-*α; Figure 3H) and interleukin-1 beta (*il-1*β; Figure 3I) were both significantly increased among mono-associated and CONR zebrafish. However, mono-associated zebrafish did not activate the expression of serum amyloid a (*saa*) gene compared with GF zebrafish but CONR zebrafish did (Supplementary Figure S3A). The CONR zebrafish had similar expression of complement factor b (*bf*) gene with 4E zebrafish, while they had higher expression than GF zebrafish, 2E, 5E, and 6E mono-associated zebrafish (Supplementary Figure S3B). Conversely, the CONR zebrafish had lower expression of myeloid differentiation factor 88 (*myd88*) gene than GF and mono-colonized zebrafish, while mono-associated zebrafish upregulated the expression of *myd88* gene than GF zebrafish except the 2E concentration (Supplementary Figure S3C).

Apparently, microbiota colonization (CONR) significantly downregulated the transcription level of the three measured stress related genes compared with GF zebrafish and mono-associated zebrafish. Interestingly, colonization of the bacteria by using the higher concentrations including 5E and 6E, decreased the expression levels of heat shock 70 (*hsp70*; Figure 3J) and x-box binding protein 1 (*xbp1*; Figure 3K), while lower concentrations (2E, 3E, and 4E) did not. The GF and mono-associated zebrafish had similar expression levels of the activating transcription factor 6 gene (*atf6*) (Figure 3L).

### The Host Responses to Different Concentrations of *B. subtilis* WB800N

**FIGURE 4:**
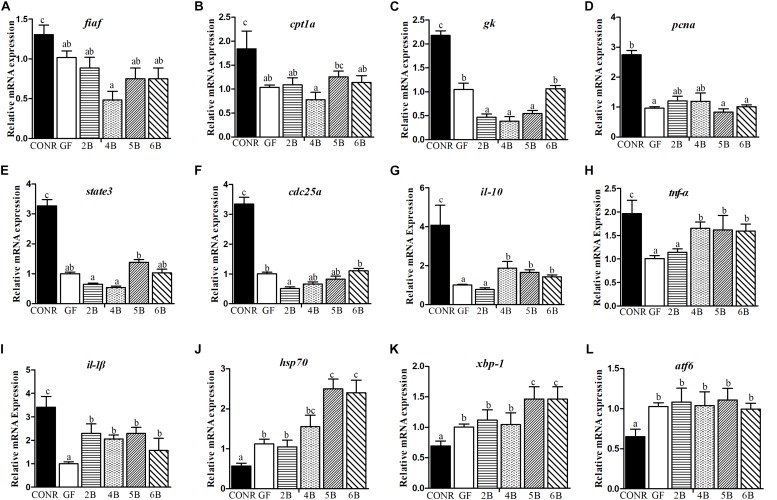
Comparisons of gene expression levels among germ-free (GF) zebrafish, CONR zebrafish and mono-associated (GF) zebrafish with different concentrations of *B. subtilis* WB800N. **(A)** The expression levels of nutrients metabolism related genes *fiaf*, **(B)** *cpt1a*, **(C)** *gk*. **(D)** The expression level of cell proliferation related genes *pcna*, **(E)** *state3*, **(F)** *cdc25a*. **(G)** The expression levels of innate immune related genes *il-10*, **(H)** *tnf-*α and **(I)** *il-1*β. **(J)** The expression levels of damage stress related genes *hsp70*, **(K)** *xbp1* and **(L)** *atf6*. The results were shown as mean ± SEM of three independent experiments with six replicates for each experiment, *n* = 90, per group. Different letters indicate statistically significant differences among groups (*p* < 0.05).

The mono-colonized zebrafish with *B. subtilis* WB800N did not influence the expression levels of the nutrients metabolism related genes [*fiaf* (Figure 4A), *cpt1a* (Figure 4B), and *gk* (Figure 4C)] or cell proliferation key genes [*pcna* (Figure 4D), *state3* (Figure 4E), and *cdc35a* (Figure 4F)] compared with GF zebrafish, although the expression levels of these genes were lower in GF zebrafish compared with the CONR ones. However, the mono-associated zebrafish promoted the expression levels of the immunity related genes such as [*il-10* (Figure 4G), *tnf-*α (Figure 4H), *il-*β (Figure 4I), *saa* (Supplementary Figure S4A), *bf* (Supplementary Figure S4B), and *myd88* (Supplementary Figure S4C)] relative to GF zebrafish, especially in the 4B and 5B mono-associated zebrafish. The mono-associated zebrafish with 5B and 6B increased the expression levels of *hsp70* (Figure 4J) and *xbp-1* (Figure 4K) genes compared to GF and CONR zebrafish. All the mono-associated and GF zebrafish increased the expression levels of *atf6* gene compared to CONR (Figure 4L).

### Behavioral Comparisons Between Zebrafish in the Presence and the Absence of Bacteria

**FIGURE 5:**
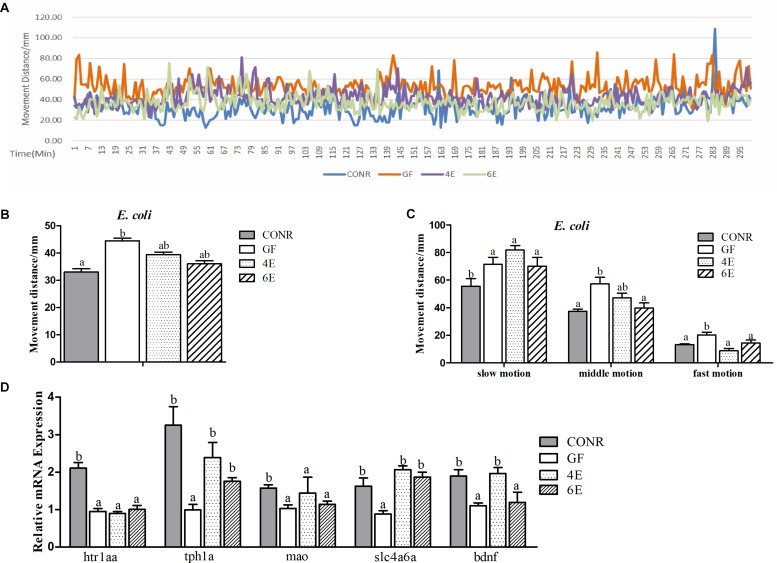
Behavior detection of CONR larvae, germ-free (GF) larvae and germ-free larvae mono-associated with 10^4^ and 10^6^ CFU/ml *E. coli* DH5α. **(A)** Traveling distance was recorded every minute, testing for 48 h in total and traveling distance during 5 h when the behavior of all zebrafish was relatively stable is shown. **(B)** Mean traveling distance of each group during the 48 h of testing. **(C)** The distance that CONR), germ-free (GF) and germ-free (GF) mono-associated zebrafish moved in slow (<8 mm/s), middle (>8 and <20 mm/s) or fast (>20 mm/s) locomotion during 48 h of testing. **(D)** The expression levels of 5-hydroxytryptamine synthesis related genes *htr1aa*, *tph1a*, *slc4a6a*, *mao*, and *bdnf*. All data **(B–D)** were presented as means ± SEM. Different letters indicate statistically significant differences among groups (*p* < 0.05).

**FIGURE 6:**
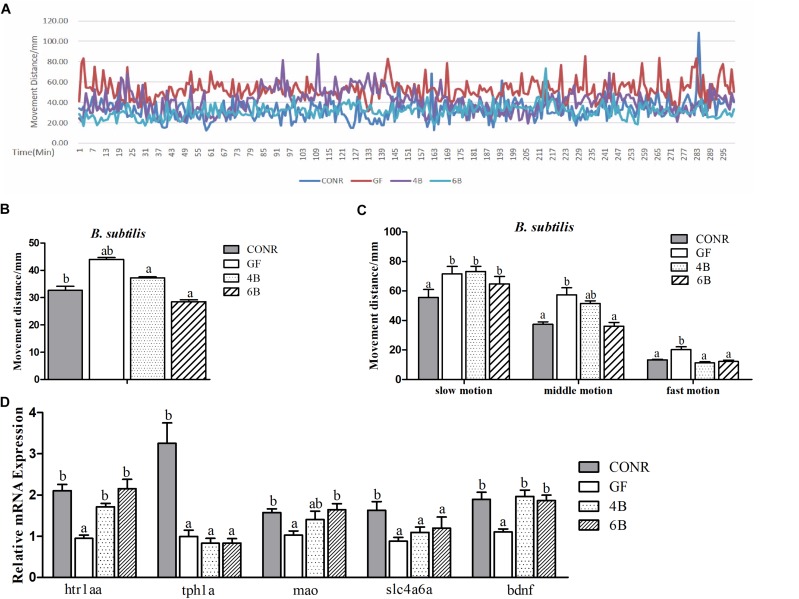
Behavior detection of CONR larvae, germ-free (GF) larvae and germ-free (GF) larvae mono-associated with 10^4^ and 10^6^CFU/ml *B. subtilis* WB800N. **(A)** Traveling distance was recorded every minute, testing for 48 h in total and traveling distance of a period of 5 h is shown. **(B)** Mean traveling distance of each group during the 48 h of testing. **(C)** The traveling distance that CONR larvae, germ-free (GF), and germ-free (GF) mono-associated zebrafish moved in slow (<8 mm/s), middle (>8 and <20 mm/s), or fast (>20 mm/s) locomotion during 48 h of testing. **(D)** The expression levels of 5-hydroxytryptamine synthesis related genes *htr1aa*, *tph1a*, *slc4a6a*, *mao*, and *bdnf*. All data **(B–D)** were presented as means ± SEM. Different letters indicate statistically significant differences among groups (*p* < 0.05).

Previous studies reported that microbial colonization are required for normal neurobehavioral development in zebrafish (Diaz Heijtz et al., 2011; Phelps et al., 2017). To demonstrate whether the different concentrations of microbial exposure influenced the neurodevelopment of zebrafish, the locomotion activities of zebrafish larvae were compared among GF, CONR and the two concentrations of the mono-associated zebrafish (10^4^ and 10^6^ CFU/ml). The results showed that, the GF zebrafish traveled more distance than CONR zebrafish (Figures 5, 6), suggesting fish in the former group were more active than the latter.

The data also revealed that the activity levels of mono-associated zebrafish for both bacteria were lower than that of GF zebrafish and higher than CONR zebrafish (Figures 5A, 6A). However, the mono-associated zebrafish with *E. coli* DH5α moved comparable distance to CONR zebrafish (Figure 5B). Conversely, the mono-associated zebrafish with *B. subtilis* WB800N decreased the distance traveled relative to the GF zebrafish, especially the 6B fish (Figure 6B). The results further showed that, the mono-associated zebrafish with either *E. coli* DH5α or *B. subtilis* WB800N, did not significantly change the slow motion but decreased the middle and fast motion significantly (Figures 5C, 6C) compared to GF fish. The different concentrations of the two bacteria species did not induce marked differences in movement.

Moreover, we measured the mRNA expression levels of brain derived neurotrophic factor (*bdnf*), 5-hydroxytryptamine receptor 1A (*htr1aa*), solute carrier family 6 member 4 (*slc6a4a*), tryptophan hydroxylase 1A (*tph1a*) and monoamine oxidase (*mao*) genes, which indicate the degree of the nervous system development. The results showed that, the expression levels of *tph1a*, *slc4a6a*, and *bdnf* were up-regulated in the existence of *E. coli* (Figure 5D), while *htr1aa, mao*, and *bdnf* were enhanced markedly in the presence of *B. sublitis* (Figure 6D). These results revealed that bacterial colonization can change behavior characteristics and promote neurodevelopment of zebrafish.

## Discussion

Gnotobiotic zebrafish is an increasingly powerful model for studying complex and dynamic relationships between intestinal microbiota and the host (Williams, 2014). Colonizing GF fish with single bacterial species (mono-association) provides opportunities for studying both the effects of bacteria on the host and its characteristics (Pham et al., 2008). The present study determined the responses of two bacteria species at different concentrations, colonization time points and incubation periods. The data demonstrated that, the final concentrations ranging from 10^4^ to 10^5^ CFU/ml guarantee high survival rates of zebrafish and effective host responses for the two bacterial species. The bacteria final concentration of 10^6^ CFU/ml is also worthy trying when the inoculation bacteria has low virulence factor or the study requires high colonization concentrations. Higher colonization concentrations may cause host damage owing to the reduced dissolved oxygen concentration in the media. These results suggest that, dissolved oxygen should be considered during GF mono-association studies. The survival rate was higher when GF zebrafish were inoculated with two bacterial strains at 3 dpf than at 5 dpf. Possibly, the zebrafish at 3 dpf were less active and thus used less dissolved oxygen compared to the zebrafish at 5 dpf, which were more developed and needed more dissolved oxygen, consistent with previous studies (Cantas et al., 2012; Jemielita et al., 2014).

The results further showed that, prolonging the exposure time to 48 h, promoted the colonization efficiency, consistent with results of a previous study (Russo et al., 2015), but our results indicated that bacteria incubation for 24 h was sufficient to induce effective host responses. A previous study on DNA microarray comparisons of gene expression in the digestive tracts of GF, conventionalized and CONR zebrafish revealed 212 genes involved in nutrients metabolism, cell proliferation, innate immunity, and neurodevelopment were regulated by the microbiota (Rawls et al., 2004). In the present study, gene expression data showed that certain colonization conditions of *E. coli* DH5α and *B. subtilis* WB800N recapitulated the host responses, which are species-specific.

The *E. coli* DH5α used in the present study mainly induced the expression of genes related to nutrients metabolism, innate immunity and cell proliferation, while *B. subtilis* WB800N mainly upregulated the expression levels of genes related to innate immunity and stress responses. Similarly, Rawls et al. (2006) revealed that *E. coli* MG1655 were able to partially recapitulate the response of lipid metabolism related genes (*fiaf, cpt1a*, and hydroxyacylCoA dehydrogenase) and innate immunity related genes (*saa* and myeloperoxidase).

It has been also proved that, the host cell proliferation responses are induced by either a single strain of bacteria or a complex microbiota community (Rawls et al., 2004, 2006; Phelps et al., 2017). In the present study, the mRNA expression levels of *pcna, state3*, and *cdc25* genes were induced by *E. coli* DH5α instead of *B. subtilis* WB800N. Our results indicated that, colonization of GF zebrafish with *E. coli* DH5α concentrations ranging from 10^4^ to 10^6^ CFU/ml induced the mRNA expression level of *pcna*, but 10^2^ CFU/ml did not. Similarly, Rawls et al. (2006) found the mRNA expression level of cell proliferation related gene (*pcna*) did not increase relative to GF zebrafish when GF zebrafish were inoculated with 10^4^ CFU/ml of *E. coli* MG1655. This result suggests that the concentrations of different bacterial strains required for eliciting host response vary between bacteria species.

The influences of bacteria species on host immunity have attracted much attention in recent years. It has been demonstrated that zebrafish innate immune development is regulated by the presence of bacteria (Galindo-Villegas et al., 2012). A previous study indicated that, CONR zebrafish activated some pro-inflammatory genes such as *il1*β, *tnf*α, *il8* (interleukin-8) and anti-inflammatory gene (*il-10*), although not all inflammatory genes responded to the presence of microbes (Galindo-Villegas et al., 2012). Moreover, *Listeria monocytogenes* induced transient expression of innate immune response genes by significantly elevating the genes related to innate immunity such as mmunoresponsive gene 1 (*irg1l*), *il1b*, and matrix metallopeptidase 9 (*mmp9*) of GF zebrafish (Shan et al., 2015). Our study showed that, the *E. coli* DH5α and *B. subtilis* WB800N at the 10^4^, 10^5^, and 10^6^ CFU/mL concentrations triggered immunity response genes such as *il1*β, *tnf*α, and *il10*. The transcription level of *myd88* is complex because of its various functions. This gene not only adjusts the innate immune setpoint as a key adaptor protein downstream the majority of TLRs, which is central in many aspects of host sensing of the microbiome (Richendrfer et al., 2012), but also controls epithelial cell proliferation. Moreover, our knowledge on the regulation of TLRs by *myd88* and its further control on downstream genes to accomplish the host responses is currently limited. A previous study reported that, intestinal microbiome adjusts the innate immune setpoint during colonization through negative regulation of *myd88* (Koch et al., 2018). However, another previous study indicated that, microbial colonization upregulated the expression level of *myd88* gene (van der Sar et al., 2006). Additionally, Qin et al. (2018) found that *Lactobacillus casei BL23* increased the expression of stress-related genes compared to GF zebrafish, suggesting the expression of these genes might be a self-protection mechanism of the host, which is consistent with our results on stress related genes.

Gnotobiotic zebrafish models have been also applied in behavior and neurodevelopment related studies (Richendrfer et al., 2012). Some studies have shown that, normal gut microbiota modulate brain development and behavior of the host including mice (Diaz Heijtz et al., 2011) and zebrafish (Davis et al., 2016). In the present behavioral study, zebrafish in the absence of microbiota showed higher levels of physical activity, consistent with axenic zebrafish, which exhibited high levels of physical activity and performed antianxiety-like behavior relative to CONR zebrafish (Richendrfer et al., 2012; Phelps et al., 2017). We also discovered that, the inoculation concentrations ranging from 10^4^ and 10^6^ CFU/ml *E. coli* DH5α and *B. subtilis* WB800N reduced host hyperactivity behavior, suggesting microbial colonization during early life is required for normal neurobehavior. More importantly, some key genes for 5-hydroxytryptamine synthesis, one of the primary neurotransmitters modulating physiological and behavioral processes in the central nervous system (Norton et al., 2008; da Rocha et al., 2019) and other genes involved in neurodevelopment of zebrafish such as *tph1a*, *mao*, *slc4a6a*, and *bdnf* were increased in the presence of the two bacteria species. The *htr1aa* gene functions as the serotonin receptor, a homolog of the mammalian serotonin receptor 1A and *slc4a6a* gene encodes serotonin transporters. The *tph1a* gene encodes tryptophan hydroxylase and is the rate-limiting enzyme in serotonin synthesis, while *mao* gene exhibits a strong affinity profile for serotonin, encoding serotonin transporters. These key genes of neurodevelopment responded to the presence of the two bacteria species at the concentrations ranging from 10^4^ and 10^6^ CFU/ml. These results indicate that, the presence of bacteria is essential for behavioral and neurodevelopment processes of zebrafish.

## Conclusion

The present study inoculated GFP-labeled *E. coli* DH5α or *B. subtilis* WB800N to GF zebrafish larvae in order to explore the responses of the host to different inoculation concentrations, colonization time points and incubation periods. We analyzed the influence of different factors on the survival rate, colonization efficiency, nutrients metabolism, stress response, innate immune, cell proliferation, and neurodevelopment. In general, our results showed that the two bacteria species at different concentrations caused species-specific responses on the host, while both bacteria species decreased the hyperactivity behavior of GF zebrafish. Therefore, determining inoculation conditions is an important aspect on GF zebrafish depending on experimental targets such as purpose, bacterial virulence, and breeding method. The inoculation conditions used in the present study provide reference information for mono-association experiments and advance the application of GF animal models in future studies.

## Data Availability

All datasets generated for this study are included in the manuscript and/or the Supplementary Files.

## Ethics Statement

The animal study was reviewed and approved by the East China Normal University (ECNU) (No. F20140101), Shanghai, China.

## Author Contributions

FT, Z-YD, and MZ designed the study. FT, FQ, and YQ executed the experiments. FT, SL, Z-YD, and MZ analyzed the data. FT drafted the manuscript. FT, SL, FQ, YQ, Z-YD, and MZ wrote the manuscript and read and approved the final manuscript for submission.

## Conflict of Interest Statement

The authors declare that the research was conducted in the absence of any commercial or financial relationships that could be construed as a potential conflict of interest.
