# Supplementary material for: The Responses of Germ-Free Zebrafish (Danio rerio) to Varying Bacterial Concentrations, Colonization Time Points, and Exposure Duration
Source: Front Microbiol. 2019 Sep 18;10:2156. doi: 10.3389/fmicb.2019.02156 (PMC6760068; doi:10.3389/fmicb.2019.02156)
Supplement: Supplementary file 1 [file Data_Sheet_1.docx]

The Responses of Germ-Free Zebrafish (*Danio rerio*) to Varying Bacterial Concentrations, Colonization Time Points and Exposure Duration

Fang Tan^1^, Samwel Mchele Limbu^1,2^, Ye Qian^1^, Fang Qiao^1^, Zhen-Yu Du^1^*, Meiling Zhang^1^*

^1^Laboratory of Aquaculture Nutrition and Environmental Health, School of Life Sciences, East China Normal University, Shanghai, 200241, China

^2^Department of Aquatic Sciences and Fisheries Technology, University of Dar es Salaam, P.O. Box 35064, Dar es Salaam, Tanzania

*** Correspondence:**

Zhen-Yu Du

Email: [zydu@bio.ecnu.edu.cn](mailto:zydu@bio.ecnu.edu.cn) Tel/Fax: 86-21-54345354

Meiling Zhang

Email: [mlzhang@bio.ecnu.edu.cn](mailto:mlzhang@bio.ecnu.edu.cn) Tel/Fax: 86-21-54345354

Running title: Bacteria mono-association of germ-free zebrafish

| Gene name | Forward (5′-3′) | Reverse (5′-3′) | Sequence Numbers |
| --- | --- | --- | --- |
| In zebrafish (*Danio rerio*) | | |  |
| **Elongation factor 1α (*ef-1α*)** | CTGGAGGCCAGCTCAAACAT | ATCAAGAAGAGTAGTACCGCTAGCATTAC | FJ915061.1 |
| **Glyceraldehyde-3-phosphate dehydrogenase(*gapdh*)** | CGCTGGCATCTCCCTCAA | TCAGCAACACGATGGCTGTAG | NM_213094.2 |
| **Bata-actin(*β-actin*)** | CGAGCTGTCTTCCCATCCA | TCACCAACGTAGCTGTCTTTCTG | XM_028814403.1 |
| **Signal transducer and activator of transcription 3(*state3*)** | TAACCTCTTACTCATCCTCCA | AAGAGGTTGTAGAAGTAGA | CR847978.18 |
| **Cell division cycle 25A (*cdc25a*)** | TCGCTCTCCTGCCTTCAAGA | GACAGCGAATGACAGGCGAA | EU921896.1 |
| **Fasting-inducing adipose factor (*fiaf*)** | CGAGCGCATCAAGCAACA | TCGCTCGTTTTTCATCGTAATCT | NM_001256203.1 |
| **Carnitine palmitoyltransferase 1a (*cpt1a*)** | GCAGATGACGGTTATGGTGTTTC | CCGTCTCAGGACTGGAGAACTT | NM_001005940.1 |
| **X-box binding protein 1 (*xbp-1*)** | TGCGAGACAAGACGAGTGAT | CAGTACCTGAACCTGCTCCTT | BC164779.1 |
| **Activating transcription factor 6 (*Atf6*)** | CTTCAGACGCCTCAGTCAATG | GACCACAGGAGATGTTGTTACC | NM_001110519.1 |
| **Serum amyloid a (*saa*)** | CGCAGAGGCAATTCAGAT | CAGGCCTTTAAGTCTGTATTTGTTG | NM_001005599.1 |
| **Complement factor b (*bf*)** | GCCACAGTGCTACGCTGATTT | GTTGAACTGTTAGAGTTGTCGTTAGAGAATT | XM_021468751.1 |
| **Proliferating cell nuclear antigen (*pcna*)** | GATGGTAGTTTGGGCCTTAGCTTT | AAAAACAAGACTGAATGTGGAGTATGTAC | NM_131404.2 |
| **Heat-shock protein (*hsp70*)** | ACCAGGGCAACAGAACAACA | GTCATCAAACCTCCTGCCGA | NM_001113589 |
| **Myeloiddifferentiationfactor88 (*myd88*)** | TCACGTACCTGGAGATCAAAAACTTCGAG | CCACTGGAACCTGAAGCGGTTTCCTC | NM_212814.2 |
| **Solute carrier family 6 member 4 (*Slc4a6a*)** | AGTGGACCTGGGCAATG | AGAAGATACGGCAAGAGAAG | DQ285098 |
| **Monoamine oxidase (*mao*)** | GCAGTCAGAGCCCGAATC | CACACCCATAAACTTGAGGAATC | NM_212827 |
| **Glucokinase (*gk*)** | GAGAGGACTGCGTGTGGAGACA | TCACCAACCTCGGAGCCTTCAG | NM_001045385.2 |
| **5-hydroxytryptamine (serotonin) receptor 1A**  **(*htr1a*)** | AGAGCAGCGAGGTGAC | GAGCCGATGATTTGGTAAC | EH441641 |
| **Tryptophan hydroxylase 1a (*tph1a*)** | CAGTTCAGTCAGGAGATTGG | GACAGTGCGTGCTTCAG | AF548566 |
| **Brain derived neurotrophic factor (*bdnf*)** | GCTCAGTCATGGGAGTCC | ATAGTAACGAACAGGATGG | U42489 |
| **Interleukin-10 (*il-10*)** | ATTTGTGGAGGGCTTTCCTT | AGAGCTGTTGGCAGAATGGT | NM_001020785.2 |
| **Interleukin-1β (*il-1β*)** | GAGACAGACGGTGCTGTTTA | GTAAGACGGCACTGAATCCA | NM_212844.2 |
| **Tumor Necrosis Factor α （*tnf-α*）** | CAGAGTTGTATCCACCTGTTA | TTCACGCTCCATAAGACCCA | NM_212859.2 |

**Supplementary Table S1.**

The primer sequences used for qPCR in the present study

**Supplementary Table S2.**

The results of normality test for measured genes in the present study

|  | ***E. coli*** | | | ***B. subtilis*** | |
| --- | --- | --- | --- | --- | --- |
| **Genes** | | **Statistic** | **P-value** | **Statistic** | **P-value** |
| *GK* | | 0.951 | *0.812 | 0.971 | *0.970 |
| *state3* | | 0.722 | **0.002 | 0.807 | 0.838 |
| *cdc25a* | | 0.983 | 0.103 | 0.875 | 0.565 |
| *xbp-1* | | 0.862 | 0.851 | 0.943 | *0.365 |
| *atf-6* | | 0.981 | 0.314 | 0.932 | *0.138 |
| *bf* | | 0.955 | 0.339 | 0.9221 | **0.021 |
| *pcna* | | 0.728 | *0.052 | 0.921 | *0.137 |
| *hsp70* | | 0.985 | 0.078 | 0.956 | 0.606 |
| *myd88* | | 0.864 | 0.596 | 0.790 | **0.014 |
| *saa* | | 0.981 | *0.877 | 0.931 | 0.055 |
| *fiaf* | | 0.953 | 0.166 | 0.784 | 0.388 |
| *cpt1a* | | 0.724 | *0.064 | 0.710 | *0.098 |
| *il-10* | | 0.987 | *0.061 | 0.871 | *0.461 |
| *tnf-a* | | 0.860 | 0.682 | 0.986 | 0.684 |
| *il-1b* | | 0.981 | 0.081 | 0.761 | 0.304 |
| *htr1aa* | | 0.953 | 0.282 | 0.954 | 0.282 |
| *tph1a* | | 0.723 | **0.006 | 0.721 | *0.073 |
| *mao* | | 0.986 | 0.861 | 0.985 | *0.858 |
| *slc4a6a* | | 0.862 | *0.491 | 0.863 | *0.491 |
| *bdnf* | | 0.981 | 0.816 | 0.981 | 0.816 |

Numbers without a symbol for p values were normally distributed (p > 0.05). The symbol * indicates the results of p values for data which were normally distributed after log transformation, while ** shows the p values results of parameters that were not normally distributed even after log transformation.

**Supplementary Figure S1.**


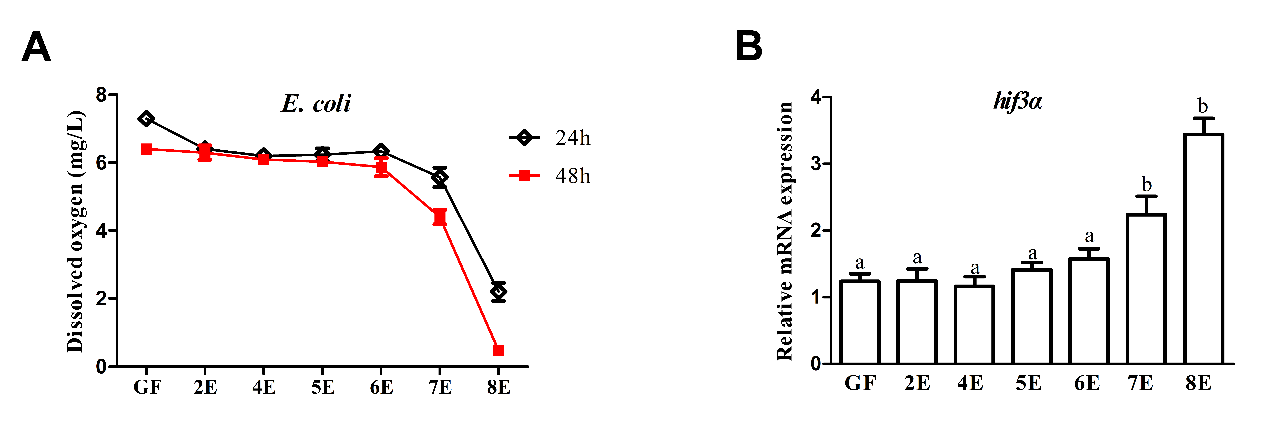


Dissolved oxygen level in the media and the expression level of hypoxia inducible factors 3α (*hif3α*) when zebrafish were inoculated with *E. coli* DH5α*.* (**A**) Dissolved oxygen level of media of GF group and all mono-associated groups (inoculated with *E. coli* DH5α ranging from 10^2^ CFU/ml to 10^8^ CFU/ml at 3 dpf and immersed for 24 h or 48 h). (**B**) The expression level of *hif3α* of GF group and all mono-associated groups (inoculated with *E. coli* DH5α ranging from 10^2^ CFU/ml to 10^8^ CFU/ml at 3 dpf and immersed for 24 h). All data (A, B) are presented as means ± SEM, n = 45 per group.

**Supplementary Figure S2.**


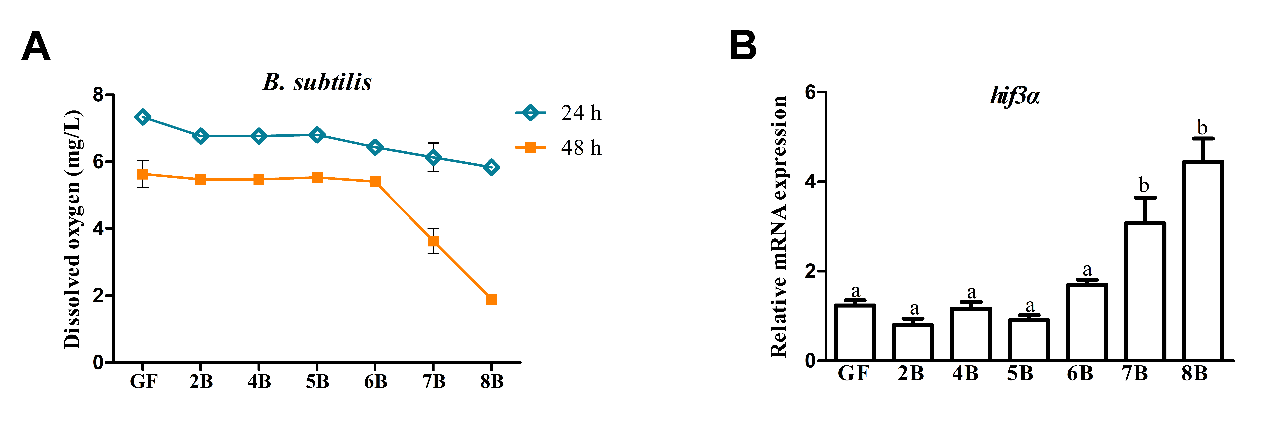


Dissolved oxygen level in the media and the mRNA expression level of hypoxia inducible factors 3 alpha (*hif3α*) when zebrafish were inoculated with *B. subtilis* WB800N*.* (**A**) Dissolved oxygen level of media of GF group and all mono-associated groups (inoculated with *B. subtilis* WB800N ranging from 10^2^ CFU/ml to 10^8^ CFU/ml at 3 dpf and immersed for 24 h or 48 h). (**B**) The expression level of *hif3α* of GF group and all mono-associated groups (inoculated with *B. subtilis* WB800N ranging from 10^2^ CFU/ml to 10^8^ CFU/ml at 3 dpf and immersed for 24 h). All data are presented as means ± SEM, n = 45 per group.

**Supplementary Figure S3**


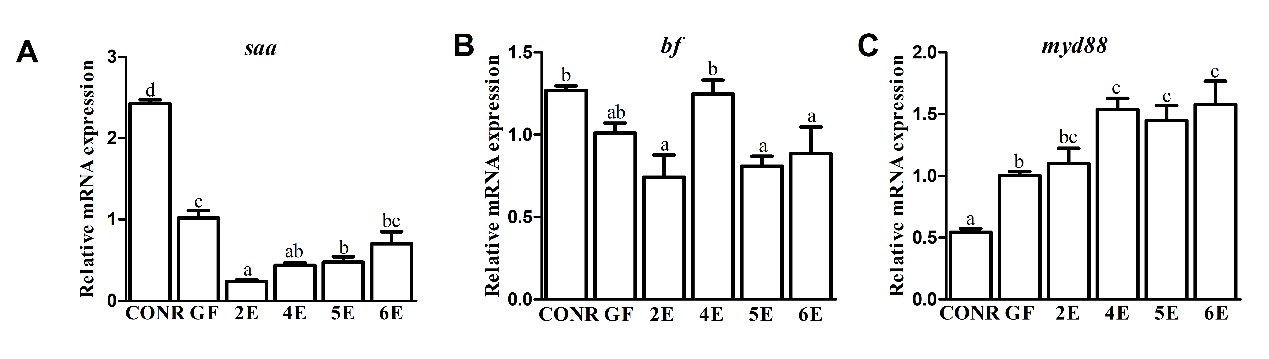


Comparisons of gene expression levels among GF zebrafish, CONR zebrafish and mono-associated zebrafish (inoculated with *E. coli* DH5α ranging from 10^2^ CFU/ml to 10^6^ CFU/ml at 3 dpf and immersed for 24 h). (**A**) The expression levels of immunity related genes *saa*, (**B**) *bf* and (**C**) *myd88.* All results are shown as means ± SEM of six replicates for each experiment, n = 90, per group. Different letters indicate statistically significant differences among groups (p < 0.05).

**Supplementary Figure S4.**


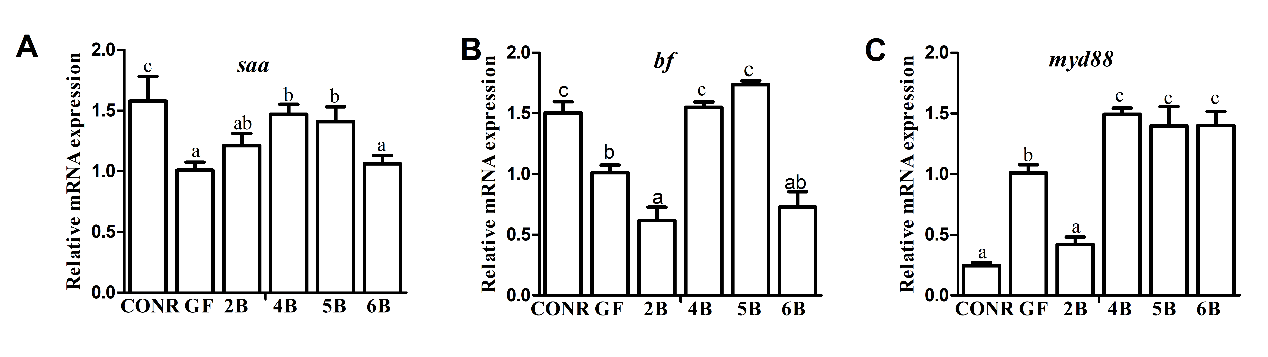


Comparisons of gene expression levels among GF zebrafish, CONR zebrafish and mono-associated zebrafish (inoculated with *B. subtilis* WB800N ranging from 10^2^ CFU/ml to 10^6^ CFU/ml at 3 dpf and immersed for 24 h). **(A)** The expression levels of immunity related genes *saa*, **(B)** *bf* and **(C)** *myd88*. The results are shown as means ± SEM of six replicates for each experiment, n = 90, per group. Different letters indicate statistically significant differences among groups (p < 0.05).
